# Olfactory receptor gene abundance in invasive breast carcinoma

**DOI:** 10.1038/s41598-019-50085-4

**Published:** 2019-09-24

**Authors:** Shirin Masjedi, Laurence J. Zwiebel, Todd D. Giorgio

**Affiliations:** 1 Department of Biomedical Engineering, Vanderbilt University, Nashville, USA; 2 Department of Biological Sciences, Vanderbilt University, Nashville, USA; 3 Department of Chemical and Biomolecular Engineering, Vanderbilt University, Nashville, USA

**Keywords:** High-throughput screening, Genome informatics

## Abstract

Expression of olfactory receptors (ORs) has been reported in many human tissues outside the nasal epithelium. ORs have been validated as biomarkers in prostate cancer. In breast cancer, however, the expression and role of OR genes remain understudied. We examined the significance of OR transcript abundance in a large invasive breast carcinoma population and identified two OR genes, OR2W3 and OR2B6 to be potentially correlated to breast cancer progression. 960 breast invasive tumors and 56 human breast cancer cell lines were assessed for OR gene expression and 21 OR genes were highly abundant among 198 cases. Our transcriptome analysis discovered three significantly abundant OR genes among three sub-populations of invasive breast carcinoma patients. OR2W3 was correlated with invasion genes and basal-like subtype whereas OR2B6 was correlated with proliferation genes and luminal A subtype. Analyzing the OR gene upregulation among breast cancer cell lines showed that OR2B6 and OR2W3 were abundant similar to invasive breast tumors. Our study suggests that specific OR genes may be correlated with breast cancer characteristics, making ORs potential new diagnostic, and/or treatment markers. This study suggests future directions for the exploration of a role for ORs in the mechanisms of breast cancer proliferation and progression.

## Introduction

Invasive breast cancer is a heterogenous disorder with phenotypic changes in tumor cells that promote progression and metastasis. Breast cancer is the second cause of death from cancer among women and the 5-year survival rate in women with metastatic breast cancer is 27%^1^. Breast cancer progression is a dynamic process influenced by the tumor microenvironment where resident cells exhibit significant alterations in their genetic expression^2,3^. The genetic alterations in tumor cells during malignant transformation promote ectopic expression of normally silent genes in tumors with potential oncogenic mechanisms^4^; cryptic olfactory receptor (OR) gene expression in tumors is an example of these ectopic expressions. Genomic analysis of mammary tumors provides extensive information that has been used to identify significantly altered genes and their interactions with the signaling pathways that correlate with immune cell infiltration, tumor cell invasion, or proliferation in breast tumors^5^. Next generation sequencing (NGS) has advanced our capabilities in the discovery of genes with potential roles in breast cancer by providing detailed genomic data from a large number of samples that is suitable for data informatics exploration, as we have carried out in this study.

ORs are the largest gene family in humans with 408 active coding genes and more than 600 pseudogenes^6,7^ identified to date. In non-human vertebrates the OR gene spectrum is considerably broader driving the highly developed sense of smell that is characteristic of canine, rodents and other animals. In the human genome, OR genes have a unique genetic structure such that they are all intron-less and polymorphic^8,9^ and are distributed across 21 of the 23 chromosomes, classified into 18 subfamilies. As to be expected, ORs are principally expressed in dendritic and axonal membranes of olfactory sensory neurons (OSNs) that populate the nasal epithelium as well as the dorsal region of the olfactory bulb^9^. ORs are G-protein coupled receptors (GPCRs) that, when activated by their corresponding molecular agonists, initiate OSN signal transduction cascades that facilitate olfactory sensitivity and perception. Beyond their role in olfaction, a handful of ORs are involved in biological processes in tissues outside the nasal cavity. For example, ectopically expressed OR1D4 has roles in mediating human sperm chemotaxis^10^ and OR51E2 is expressed in smooth airway muscle cells where it modulates cytoskeletal remodeling when activated by its endogenous ligands, acetate and propionate^11^. Recently, several ORs have been shown to play roles in cancer proliferation or progression in a few cancer types. Indeed, OR51E2 and OR51E1 have been reclassified in human prostate cancer cells as prostate specific GPCRs (PSGR and PSGR2 respectively). OR51E2/PSGR activation with β-ionone has been shown to inhibit cell proliferation^12^ and prostate tumor xenografts expressing PSGR generate larger tumors in mice compared to normal prostate tumors^13^. OR7C1 has been identified as a novel functional marker in colorectal cancer and has been implicated as a potential target for immunotherapy^14^. In gastrointestinal neuroendocrine carcinoma, six novel cancer markers have been identified in enterochromaffin cells, one of which is OR51E1^15^. In breast cancer, patients carrying mutations in the checkpoint kinase-2 (CHEK2) gene, which is a moderate penetrance breast cancer risk gene, 34 out of 144 elevated genes were OR genes^16^. A study on high-fat diet fed mice revealed co-upregulation of breast cancer-related and OR genes among the top upregulated genes during the development of obesity, a potential risk of breast cancer^17^. Transcript abundance of OR2B6 was identified in breast tumors from seven patients with ductal carcinoma^18^. Previous attempts in identifying the role of ORs in cancer have validated the expression of one or a few ORs in a specific tumor type or cells. A comprehensive genomic analysis of OR genes among a large breast cancer patient population has not been previously reported. This study investigates OR transcript abundance among human invasive breast carcinoma cases.

In this study, we characterized OR upregulation in invasive breast cancer patients by determining the level of transcript abundance and DNA amplification of these genes. Using The Cancer Genome Atlas (TCGA) breast invasive carcinoma study and the cancer cell line encyclopedia (CCLE), we analyzed the abundance of OR gene expression for each and the presence of shared OR genes among invasive breast carcinoma cases and human breast cancer cell lines. Availability of both RNAseq data and DNA copy number alterations across a large patient population, along with clinical survival data, makes TCGA an ideal data source for our comparative investigation. RNAseq data of invasive breast carcinoma cases were obtained and upregulation of ORs and some major breast-cancer related genes was calculated to identify the significantly abundant OR genes. The simultaneous abundance of breast cancer-related genes and OR genes opens the possibility of ORs as key role-players in breast cancer.

## Results

### Less than 5% of OR genes are potentially significant in breast cancer

**Figure 1:**
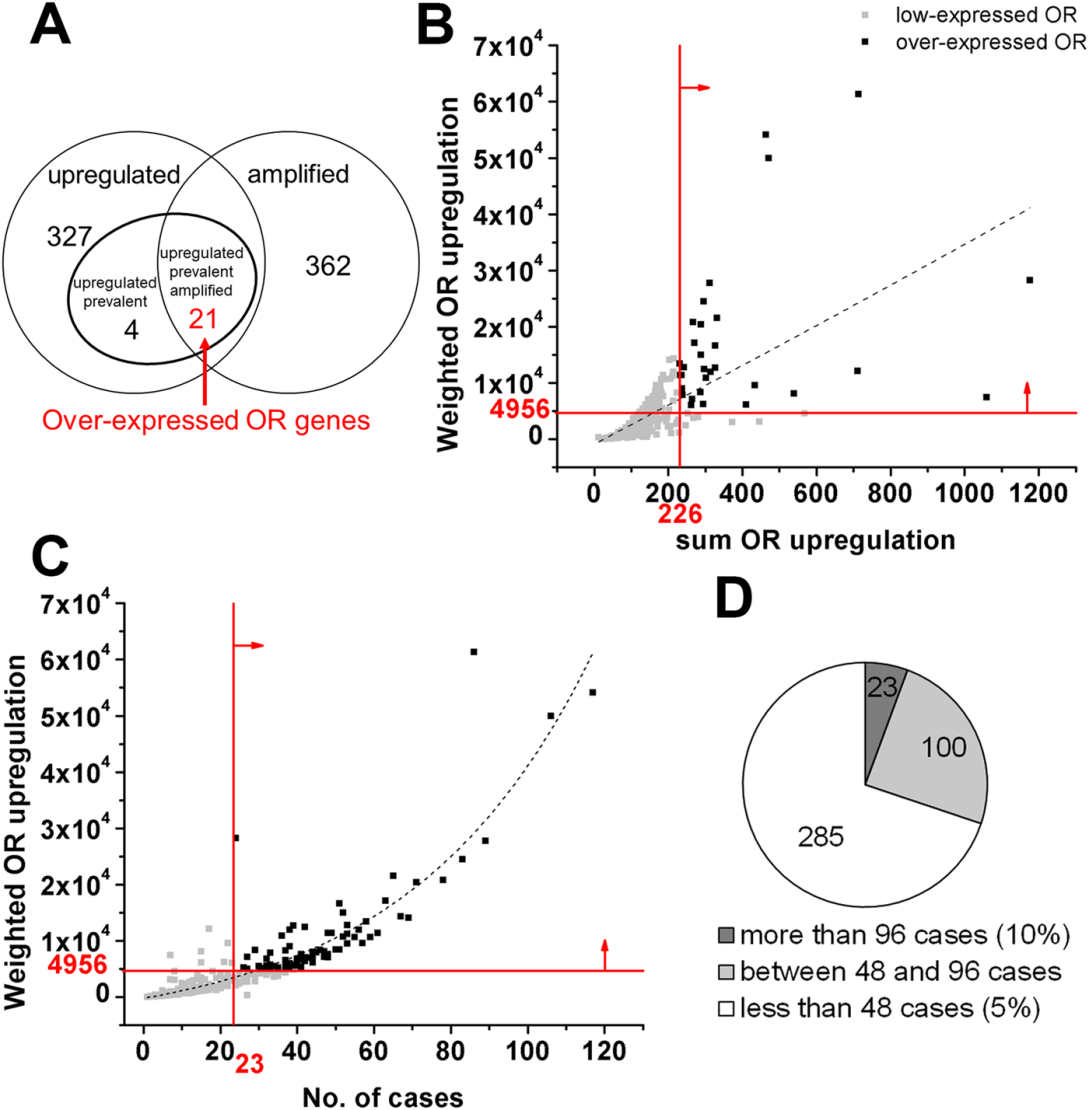
Algorithm for identifying the potentially significant OR genes among breast invasive carcinoma patients. (**A**) By considering the amplification levels, upregulation and prevalence, 21 OR genes were identified as over-expressed ORs. (**B**) 32 OR genes exceeded the sum of OR upregulation and weighted OR upregulation thresholds of significance. (**C**) 92 OR genes exceeded the number of cases with upregulated ORs and weighted OR upregulation thresholds of significance. (**D**) Only 23 OR genes were expressed in more than 10% of the patient population, whereas the majority of OR genes were expressed in less than 5% of the population.

To identify the potentially significant OR genes among the invasive breast carcinoma cases, an algorithm was applied that identified 21 upregulated OR genes (<5% of ORs) with high-level chromosomal copy number (Fig. 1A). Our two-part algorithm combined transcript abundance level, DNA amplification and prevalence by selecting the OR genes that are 1) significantly upregulated, 2) highly amplified and 3) prevalent among breast carcinoma patients. We defined the intersection of these ORs as “over-expressed OR genes”.

In the first part of the algorithm, the thresholds of significance for sum OR upregulation and weighted OR upregulation were computed as 226 and 4956 respectively (p < 0.05). 32 OR genes exceeded both thresholds of significance indicating a significantly elevated level of upregulation compared to the other OR genes (Fig. 1B). In the second part of the algorithm, computing the thresholds of significance for the number of cases with OR upregulation and weighted OR upregulation resulted in 23 and 4956, respectively. Similarly, 92 OR genes exceeded both thresholds of significance indicating prevalence in upregulated OR genes (Fig. 1C). By comparing the two sets of data, 25 OR genes were shared indicating both significant prevalence and upregulation.

The DNA amplification level corresponding to OR genes in breast invasive cancers may suggest involvement in tumor progression through signaling deregulation and/or mechanistic roles in breast cancer. 383 OR genes were highly amplified in one or more breast carcinoma patients. By comparing the highly amplified OR genes with the 25 OR genes previously identified as significantly upregulated and prevalent, it was found that 21 OR genes show significant upregulation, high-level amplification and high prevalence, as shown in Fig. 1A. The other four OR genes that were upregulated and prevalent among a great number of cases, were not highly amplified in the tumors and were therefore not included in the “over-expressed OR genes”. This is the first identification of OR genes in human breast tumors that are upregulated and amplified in more than 12% of the study population.

Similar analysis was performed to examine the downregulation of OR genes in the breast tumors and resulted in no OR gene with significant downregulation, high-level amplification and prevalence among invasive breast carcinoma patients.

352 out of 408 OR genes initially demonstrated upregulation with greater than 2-fold change (z-score) among 960 invasive breast carcinoma patients. For 285 of the OR genes, the upregulation occurred in fewer than 48 cases (<5% of the total study population). However, 23 OR genes were upregulated in more than 96 cases (>10% of the study population) and the 21 over-expressed OR genes in more than 115 cases (>12% of the study population) indicating greater prevalence (Fig. 1D).

### OR2B6, OR2W3, OR2T8 are significantly upregulated among invasive breast carcinoma patients

**Figure 2:**
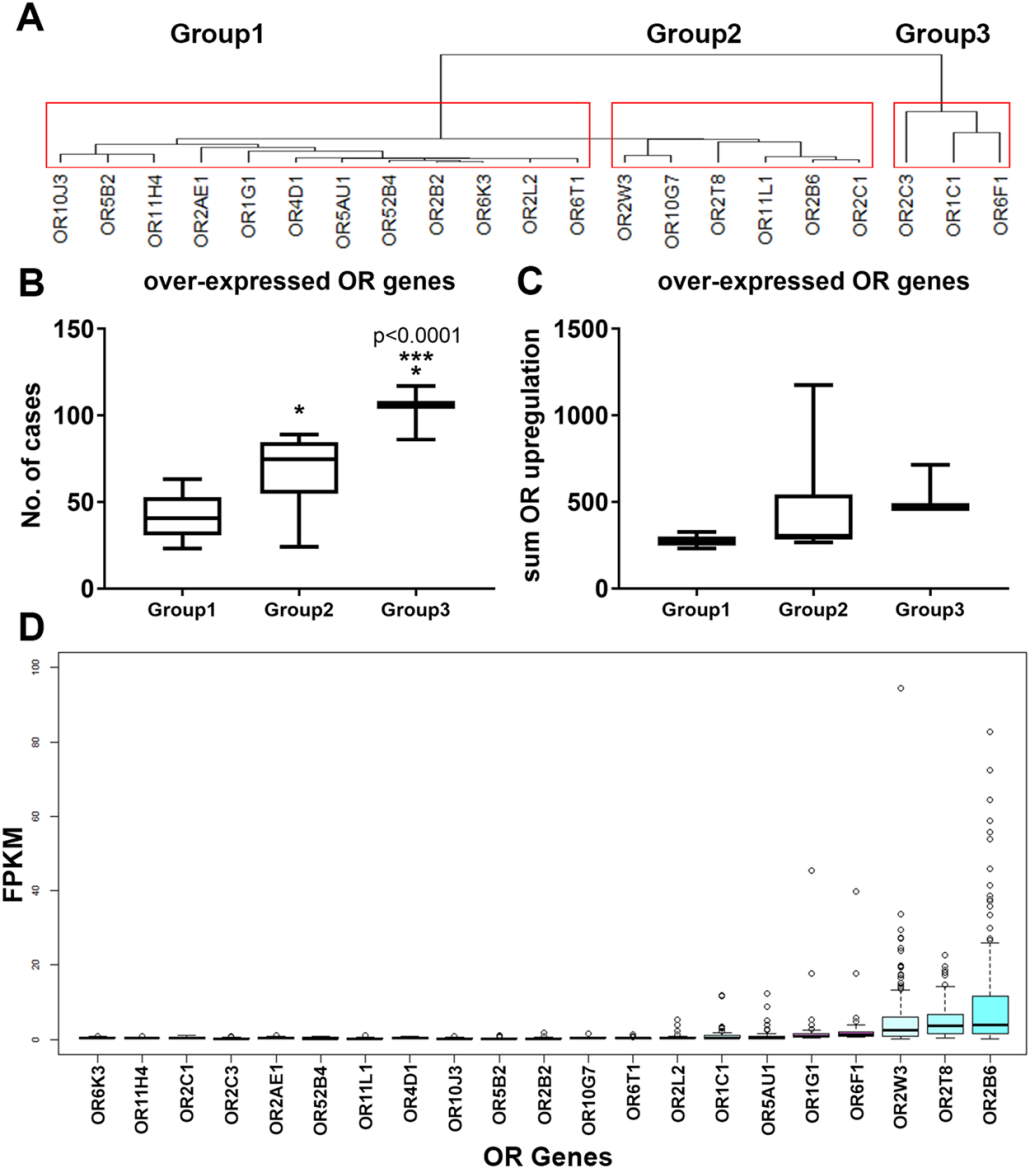
(**A**) Hierarchical clustering of the 21 over-expressed ORs based on their level of transcript abundance in breast carcinoma patients showed three distinct OR gene groups. (**B**) Number of cases with upregulated OR in each of the OR groups showed that ORs from group 3 had a significantly greater number of cases compared to group 1 and group 2 ORs (***p < 0.0001). (**C**) Sum of OR upregulation in ORs from group 3 were comparable in all OR groups (*p < 0.01, one-way ANOVA). (**D**) By considering the amplification of OR genes in breast carcinoma patients, the highest upregulation levels were observed in OR2B6, OR2T8 and OR2W3.

The potential for OR gene(s) as breast invasive carcinoma markers was evaluated by analyzing the upregulation trends in the over-expressed OR genes. First, hierarchical clustering of the 21 over-expressed OR genes based on their abundance among all the 960 patients in the study identified three groups of OR genes among the over-expressed ORs (Fig. 2A). The OR genes that are classified in “Group3” had the greatest number of cases out of 960 patients with OR gene upregulation compared to “Group1” and “Group2”, and similarly “Group2” ORs had greater number of cases than “Group1” (Fig. 2B). However, the sum of OR upregulation levels was not significantly different among the three classified OR groups (Fig. 2C). These observations indicate that there are a few OR genes that are significantly more prevalent among the breast carcinoma patients; however, the total level of upregulation for these OR genes in all the patients is not different. Based on the amplification levels of OR genes in each patient, 198 breast carcinoma cases possess significant upregulation in at least one the 21 over-expressed OR genes. The greatest level of upregulation in these 198 patients were observed in OR2W3, OR2T8 and OR2B6, all in Group2 (Fig. 2D). These results confirm the potency of our algorithm of combining amplification and upregulation to find a potential significant OR gene in invasive breast cancer.

OR gene mutation occurred in 31% of the breast invasive carcinoma patients. Table 1 lists the 15 OR genes with mutations and the number of cases in which they were both mutated and upregulated. None of the mutated OR genes were among the 21 over-expressed OR genes, suggesting that the upregulation of the over-expressed ORs is not associated with OR gene mutation in invasive breast carcinoma patients.

**Table 1:** Mutated OR genes in invasive breast carcinoma patients.

OR genes	OR5I1	OR11H1	OR2G3	OR2T27	OR2T33	OR4A15	OR5AK2	OR5L2
No. of mutations	8	8	7	7	6	6	6	6
**OR genes**	**OR5T1**	**OR6K2**	**OR8H1**	**OR8K1**	**OR10G3**	**OR10R2**	**OR14C36**	
No. of mutations	6	6	6	6	6	6	6	

None of these mutated OR genes are among the over-expressed OR gene list.

**Figure 3:**
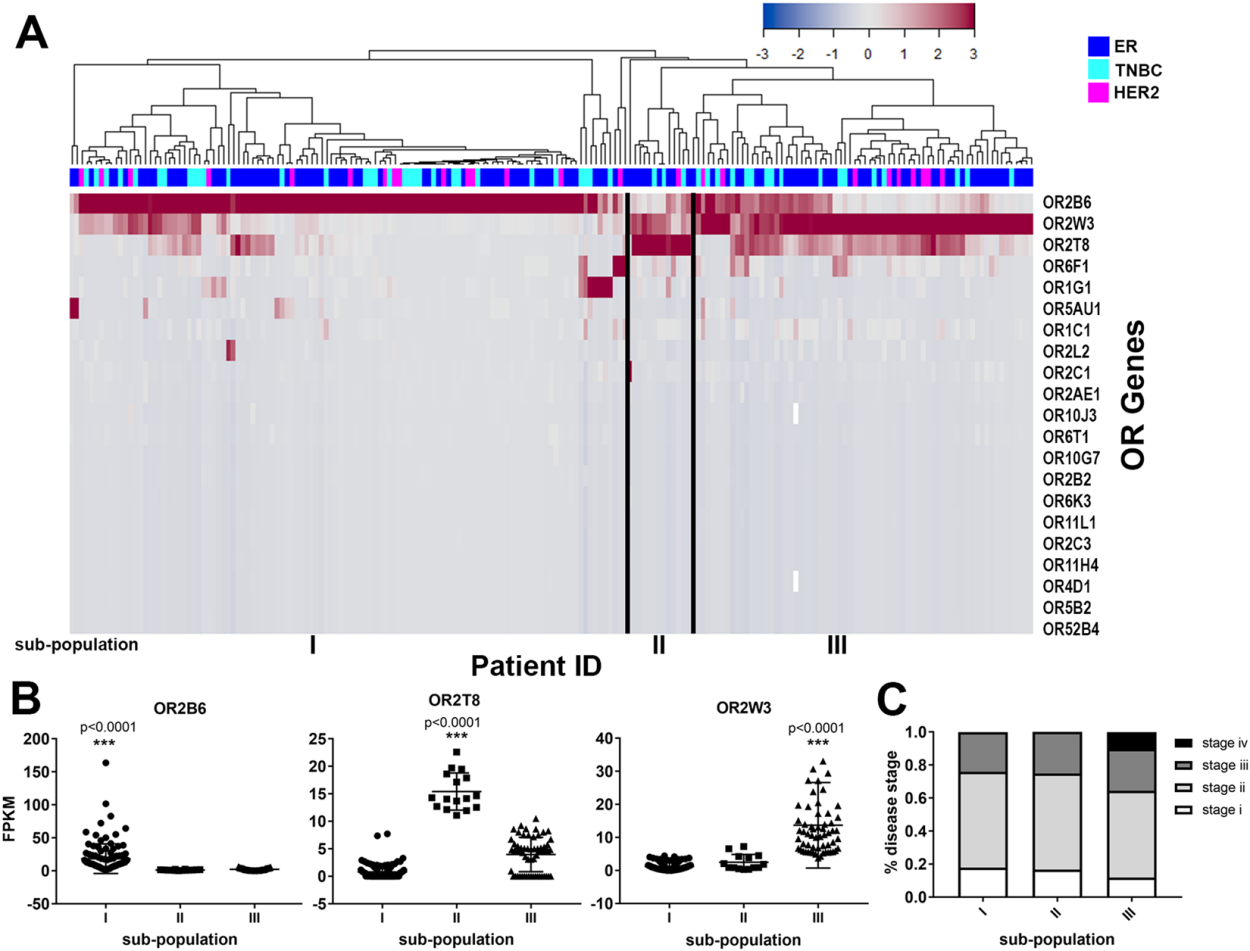
Top three OR genes expressed in each patient sub-populations. (**A**) Supervised clustering of OR transcript abundance per each patient identified three distinct sub-populations with one specific OR gene upregulation. (**B**) OR2B6 has greater transcript abundance in sub-population I, OR2T8 in sub-population II, OR2W3 in sub-populations III (***p < 0.0001, One-way ANOVA). (**C**) The majority of patients were at stage ii of breast carcinoma in all sub-populations, sub-population III was the only one with stage iv patients which correlates with cancer invasion and recurrence.

Supervised clustering of invasive breast carcinoma patients based upon abundance of the 21 over-expressed OR genes resulted in three distinct sub-populations, where one OR genes was uniquely upregulated in each of the three sub-populations (Fig. 3A). The three ORs with the highest upregulation among all the breast carcinoma patients were: OR2B6, OR2T8, and OR2W3. Cases with significant transcript abundance of OR2B6 are represented in sub-population I, where they possess significantly greater FPKM levels than other cases. Similarly, cases with abundance of OR2T8 and OR2W3 are defined as sub-populations II and III (Fig. 3B). Transcript abundance of OR6F1 and OR1G1 were also observed in a few patients but these ORs were not significant among the patient sub-populations (Fig. S2). Overall, our results suggest that specific sub-populations of invasive breast carcinoma patients have abundance of OR2B6, whereas others have abundance of OR2T8 or OR2W3.

Each sub-population of invasive breast carcinoma with abundance of OR2B6, OR2T8 or OR2W3 was characterized by breast cancer molecular subtypes, morphology and disease stages. The only sub-population with stage iv breast cancer cases was sub-population III (OR2W3 upregulation), although other disease stages among the three sub-populations were not different (Fig. 3C). There was no significant difference among the number of ER-positive, HER2-positive or triple-negative breast cancer (TNBC) cases among the three sub-populations, although slightly more TNBC cases were among the sub-population III (27%) (OR2W3 upregulation) patients compared to sub-populations I and II (25% and 23%) (OR2B6 and OR2T8 upregulation) (Fig. S3A). The tumor morphological subtype was not different in distribution among the three sub-populations of invasive breast carcinoma, where majority of cases in all sub-populations had invasive ductal carcinoma (IDC) and a mixed sub-phenotype of IDC and invasive lobular carcinoma (ILC) was observed in a few cases in sub-populations I and III (Fig. S3B).

### OR2W3 upregulation is potentially correlated with tumor invasion gene markers in invasive breast carcinoma

**Figure 4:**
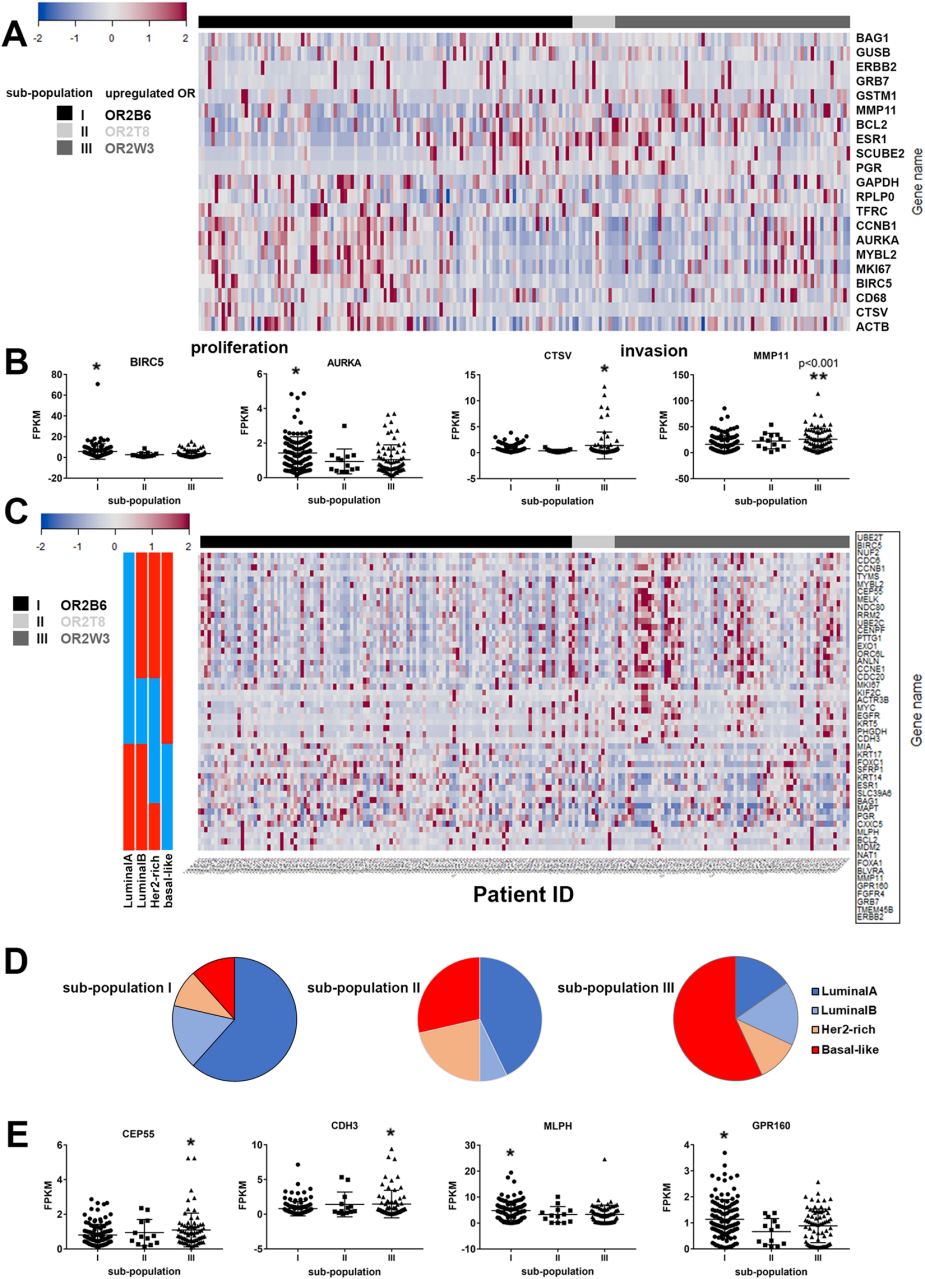
(**A**) Patterns of up or down-regulation of the 21 Oncotype DX gene panel among the invasive breast carcinoma patients. Heatmap shows the breast carcinoma sub-populations in the same order that they were clustered based on upregulation of OR2B6, OR2T8 and OR2W3. (**B**) Transcript abundance of genes associated with breast cancer proliferation were significantly greater in sub-population I with OR2B6 upregulation, and genes associated with cancer invasion were greater in sub-population III with OR2W3 upregulation. (**C**) Heatmap of PAM50 genes upregulation in invasive breast carcinoma patients, the patient sub-populations are in the same order that they were clustered based on upregulation of OR2B6, OR2T8 and OR2W3. (**D**) 60% of the sub-population III with OR2W3 matched the basal-like subtype. (**E**) Sub-population I, with OR2B6 upregulation, had significantly greater levels MLPH and GPR160, whereas sub-population III with OR2W3 upregulation had greater levels of CEP55 and CDH3 (*p < 0.05, One-way ANOVA).

Upregulated OR genes were cross-correlated with the genes in the Oncotype DX and PAM50 panels to examine the involvement of ORs in the underlying pathways that drive breast cancer proliferation or invasion. The Oncotype DX test is a genetic analysis tool commonly used in cancer prognosis and recurrence scoring in clinical settings and incorporates gene signatures correlated with cancer proliferation, invasion, hormone receptors, immune cell activation and a number of reference genes^19^. The Oncotype DX genes possessed a distinct pattern of up- or down-regulation among the three sub-populations of invasive breast carcinoma cases with specific OR gene upregulation, and more clearly between sub-populations I and III (Fig. 4A). Quantitative analysis of transcript abundance among the three sub-populations of OR genes confirmed that the genes associated with breast cancer cell proliferation (MKI67, AURKA, BIRC5, CCNB1, MYBL2) were significantly abundant in sub-population I with OR2B6 upregulation (Figs 4B and S4A–C). Genes associated with breast tumor invasion (CTSV, MMP11) were significantly abundant in sub-population III with OR2W3 upregulation compared to other sub-populations (Fig. 4B). ESR1 and ERBB2 genes (corresponding to ER and HER2 respectively) were not significantly different among the sub-populations of invasive breast carcinoma, which is in agreement with our breast cancer subtype evaluation among sub-populations with upregulated OR genes in Fig. S3A.

Similar analysis was performed on the PAM50 genes among the invasive breast carcinoma sub-populations of OR genes (Fig. 4C). PAM50 is a genetic test for identification of intrinsic subtypes of breast tumors and predicting the chemotherapy efficacy in breast cancer patients^20,21^. The majority of the cases among the sub-population I with OR2B6 upregulation matched signatures of Luminal A, whereas sub-population III with OR2W3 upregulation matched signatures of Basal-like subtypes (Fig. 4D), although cases with signatures of Luminal B or HER2-enriched subtypes were also observed in all three sub-populations of OR genes. CEP55 and CDH3 genes were significantly upregulated in sub-population III with OR2W3, indicating a correlation to Basal-like subtype. Similarly, MLPH and GPR160 genes were significantly upregulated in sub-population I with OR2B6, indicating a correlation to Luminal A subtype (Fig. 4E).

### OR2W3 is associated with decreased survival probability in invasive breast carcinoma patients

**Figure 5:**
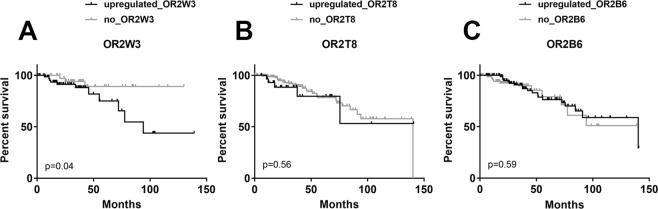
Kaplan-Meier plots of survival probability in invasive breast carcinoma patients. (**A**) OR2W3 upregulation resulted in significantly lower survival probability among invasive breast carcinoma patients (*p < 0.05, log-rank test). (**B**) OR2T8 upregulation and (**C**) OR2B6 upregulation among the invasive breast carcinoma patients did not result in lower survival probability.

OR2W3 gene upregulation significantly decreased the survival probability in invasive breast carcinoma patients to 35% after 150 months (Fig. 5A) and to nearly zero after 250 months (Fig. S5A) compared to 85% in patients with no OR2W3 upregulation. However, upregulation of OR2B6 and OR2T8 did not have any significant effect on the survival probability of the invasive breast carcinoma patients (Fig. 5B,C). Our results suggest that among the over-expressed ORs, OR2W3 may have an impact on the survival probability among invasive breast carcinoma patients.

When any of the OR genes from Group3 were expressed in invasive breast carcinoma patients, significantly less survival probability (20%) was observed compared to other patients (60%) (Fig. S5B). This may suggest that ORs from Group3, although not having the greatest upregulation levels, may be potentially significant in estimating the survival chance of breast carcinoma patients. However, when any OR genes of Group1 and Group2 were upregulated, no significant change in the survival probability of invasive breast carcinoma patients was observed. This does not contradict our OR2W3-specific results, as all the ORs from Group2 were analyzed as a cohort in this comparison.

### OR2B6 and OR2W3 are also upregulated in human breast cancer cell lines

**Figure 6:**
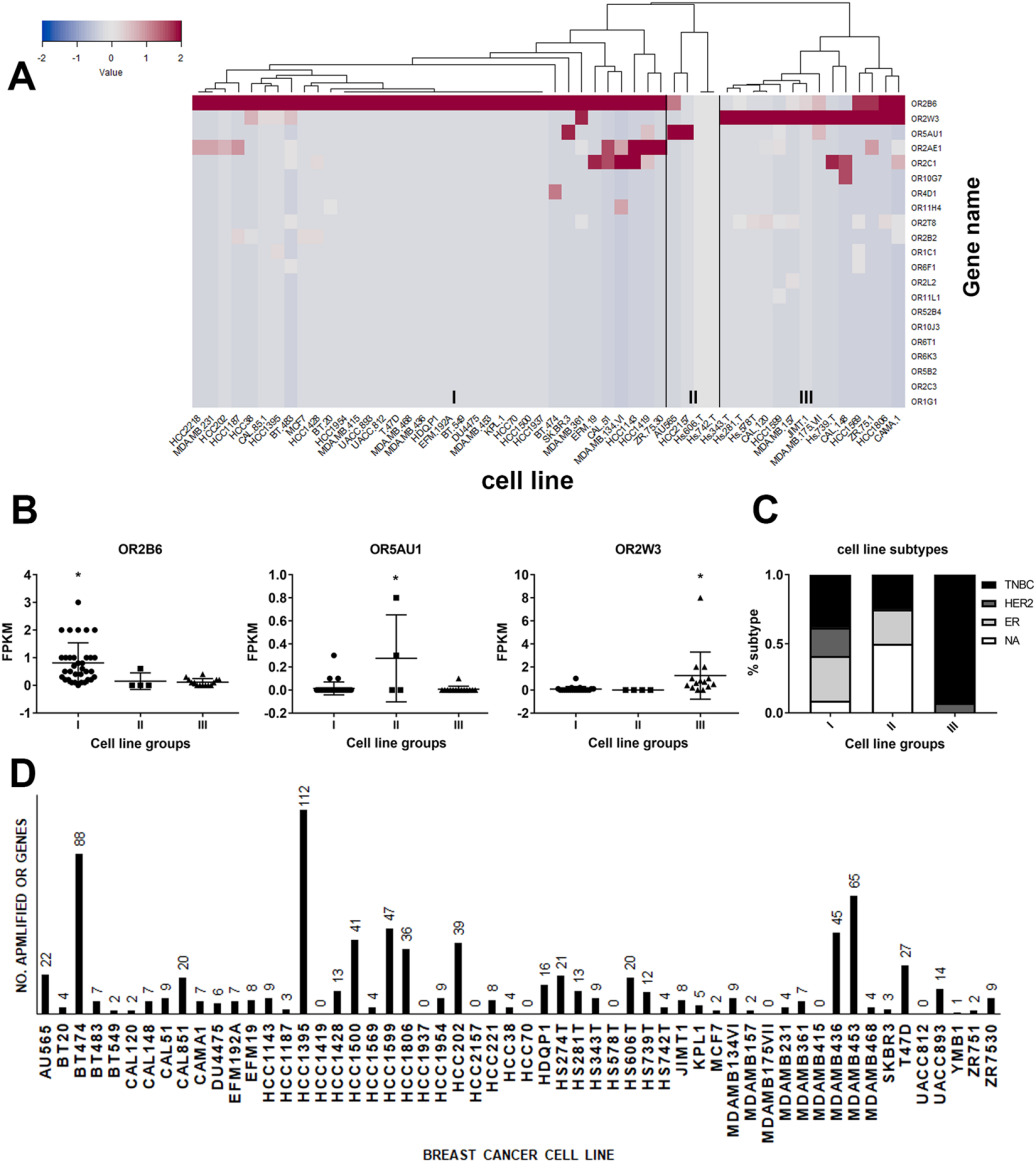
Transcript abundance of the over-expressed OR genes among 54 human breast cancer cell lines. (**A**) Clustered heatmap of human breast cancer cell lines based on their upregulation of the over-expressed OR genes demonstrated three distinct cell line groups, each with exclusive upregulation of one OR gene. (**B**) Quantitative comparison of the upregulation levels reveals that OR2B6 is significantly upregulated in cell line group I, OR5AU1 is significantly upregulated in cell line group II and OR2W3 is significantly expressed in cell line group III (*p < 0.05, One-way ANOVA). (**C**) 90% of breast cancer cell lines in group III were TNBC cell lines. (**D**) The majority of breast cancer cell lines possess high-level amplification in the OR genes with significant abundance.

54 of 56 human breast cancer cell lines were associated with significant upregulation of at least one OR. Supervised clustering of the human breast cancer cell lines based upon abundance of the over-expressed OR genes stratified the cell lines into three distinct groups (Fig. 6A). Each cell line group was characterized by significantly elevated upregulation levels of one OR gene: OR2B6, OR5AU1 or OR2W3, in groups I, II and III respectively (Fig. 6B). A comparison between the OR transcript abundance in breast carcinoma patients (Fig. 3A) and human breast cancer cell lines (Fig. 6A) identified two OR genes that were upregulated in both cases: OR2B6 and OR2W3. Table 2 lists examples of the most commonly used human breast cancer cell lines in which OR2B6 or OR2W3 were upregulated. Our own RNAseq studies have also validated OR transcript abundance in a number of the breast cancer cell lines (underlined in Table 2) and confirmed that our observation of OR upregulation in the breast cancer cell lines are comparable to the results obtained from CCLE (Fig. S6). OR2B6 upregulation level was nearly 24-fold greater in invasive breast carcinoma patients (sub-population I) than in human breast cancer cell lines (group I). Similarly, OR2W3 upregulation level was nearly 10-fold greater in invasive breast carcinoma patients (sub-population III) compared to human breast cancer cell lines (group III).

**Table 2:** Examples of human breast cancer cell lines with the highest transcript abundance of OR2B6 and OR2W3.

Cell line group	III	I
Gene	OR2W3	OR2B6
Human breast cancer cell line	ZR_75 HCC1599 MDAMB157 CAL120	BT549 MCF7 MDAMB231 MDAMB468 T47D

The underlined breast cancer cell lines were also validated in our lab for their OR upregulation.

We used the shared OR expression to correlate human breast cancer cell lines with characteristics of invasive breast cancer. More than 90% of the breast cancer cell lines in group III with OR2W3 upregulation were TNBC cell lines (Fig. 6C). TNBC characteristics were also present in group I with OR2B6 upregulation or group II with OR5AU1 upregulation, although represented by lower percentages. HER2-positive cell lines were most strongly associated with group I with OR2B6 upregulation and were excluded from group II with OR5AU1 upregulation. Cell lines with ER-positive characteristics were approximately equally divided among groups I and II and were excluded from group III. Nearly 50% of the cell lines in group II with upregulation of OR5AU1 were not classified among the most common human breast cancer phenotypes (NA). Since OR2W3 was significantly upregulated in breast cancer cell lines of group III, it may be inferred that OR2W3 is upregulated in TNBC cell lines and it may be correlated with breast cancer cell line invasiveness. The molecular characteristics of invasive breast cancer (TNBC, HER2-positive, ER-positive) in the breast cancer cell lines according to their OR gene upregulation were not similar to those observed in breast carcinoma patients, specifically between cell group I and patient sub-population I with OR2B6 upregulation, and between cell group III and patient sub-population III with OR2W3 upregulation.

High-level OR gene amplification occurred in 45 human breast cancer cell lines for at least one significantly abundant OR gene (Fig. 6D). This confirmed that upregulation and DNA amplification of one or more OR genes are present in the majority of the human breast cancer cell lines studied here that represent the majority of model systems used in breast cancer research.

## Discussion

Upregulation of OR genes have been reported in many tissues under pathological conditions, more specifically in some human cancer types. However, these reports generally examined expression of one OR gene in a few samples without providing an understanding of OR gene correlation to cancer progression supported by population-based statistics. The present work examines the abundance of an understudied gene family, ORs, among invasive breast carcinoma patients and human breast cancer cell lines. We performed a comprehensive statistical analysis on all the protein-coding OR genes among a large population of invasive breast cancer patients. This study is the first to evaluate all the OR genes in human for potential significance in invasive breast carcinoma.

Ectopic expression of OR genes has been confirmed in other human cancer types such as OR51E1 as a marker or potential therapeutic target in gastrointestinal neuroendocrine carcinoma^15^, and OR51E2 and OR51E1 in prostate cancer^12,22,23^. In fact, OR51E2 and OR51E1 have been established as prostate specific G-coupled receptor PSGR and PSGR2 among prostate cancer cells and are associated with tumor progression, cancer cell invasiveness and metastasis^24^. Identification of PSGR occurred in early 2000s before next generation sequencing technology was commonly available and the techniques were limited to blotting techniques and individual gene PCRs. PSGR was initially identified in prostate tissue from 50 samples using northern blotting^22^. For PSGR2, real-time PCR was used to demonstrate a 10-fold change compared to normal tissue^23^. In breast carcinoma, only a handful of recent studies have reported correlations between OR gene expression and invasive breast tumors. In a study on 126 patients with mutations in the checkpoint kinase-2 (CHEK2) gene, transcription of 34 OR genes was elevated^16^. Another study examined the expression of OR2B6 among 6 human breast carcinoma samples and confirmed OR2B6 protein expression in tumors by staining^18^. Our study uses RNAseq and DNA amplification data for all OR genes in human to provide a correlation between abundance of OR genes and invasive breast cancer and to find any potential significant OR gene as a specific breast cancer marker.

Advancement in the next generation technologies has enabled the identification of OR genes in various tumor samples. However, deriving meaningful information from the complex data obtained from RNAseq requires understanding of breast cancer molecular biology and biostatistics. Statistical models have been applied to minimize the bias introduced by the relative length of transcripts when computing FPKM for one gene in different samples. Some models suggested normalization methods like Trimmed Mean of M-values (TMM)^25^ to remove the bias by using factors like RNA production in raw reads instead of transcript length. Other models perform normalization after computing the read counts and are able to evaluate differential expression of genes^26^. In this study, we computed FPKM per gene per patient and used a normalization method on FPKM to identify gene upregulation based on the standard deviation (Z-score). Since the levels are normalized, the OR gene upregulations can be compared. In addition, we developed an algorithm for identifying significant ORs by combining upregulation, amplification and prevalence to only choose OR genes that are abundant in a considerable number of breast cancer patients where the corresponding gene is also amplified. Resulting over-expressed ORs among invasive breast carcinoma cases may indicate functional and/or correlative involvement in invasive breast tumors.

Genetic profiling of the tumors may provide a useful resource for discovery of the pathways associated with abnormal gene upregulation in invasive breast carcinoma. The TCGA database provides extensive detailed genomic data in a large cohort and is ideal for our comparative study. TCGA, and more specifically, the invasive breast carcinoma study, has been used for similar genetic verifications, such as understanding the correlation between DNA methylation and gene markers and/or subtypes of breast cancer^27–31^. Our results, based on statistical analysis of the TCGA invasive breast carcinoma cases, confirm that ORs are significantly expressed among a considerable fraction (>20%) of the cases. OR2B6, OR2T8 and OR2W3 were significantly upregulated in sup-populations of invasive breast carcinoma patients. Our results regarding OR2B6 abundance is in agreement with the study that reported OR2B6 protein expression in six breast carcinoma tissues^18^. Nonetheless, our data is more comprehensive as we confirmed OR2B6 upregulation in a large number of invasive breast carcinoma population and moreover, identified correlations among OR2B6 and breast cancer-related genes associated with cancer cell proliferation. OR2B6 was also correlated in this study with the Luminal A subtype that is known to be an ER-positive breast cancer. Our results also discovered that significant OR2W3 overexpression occurs in the patient sub-populations with elevated levels of breast cancer invasion-related genes and basal-like subtypes that are classified as TNBC. The highest percentage of TNBC cases and all the stage iv tumors were observed in the sub-population with OR2W3 upregulation. Moreover, OR2W3 gene expression was associated with significantly reduced probability of survival among breast carcinoma patients. These results may suggest that OR2W3 is a potential breast cancer invasion marker.

Our examination of OR gene expression in 54 breast cancer cell lines revealed significant upregulation of OR genes, and supervised clustering classified the breast cancer cell lines into three groups each exclusively expressing one OR gene. Importantly, the significant OR genes that were identified based on our methodology among the invasive breast carcinoma patients are also significantly upregulated among the breast cancer cell lines (OR2B6 and OR2W3). This indicates a potential correlation between the clinical significance of OR upregulation and the characteristics of progression and/or invasiveness of these breast cancer cell lines. The majority of the breast cancer cell lines correlated with OR2W3 are TNBC. This important outcome raises previously unknown considerations for the significance of OR2W3 in invasive breast cancers, especially TNBC subtypes and indicates the need for additional correlative and mechanistic investigations. In invasive breast carcinoma patients, tumors with OR2B6 or OR2W3 upregulation possessed comparable numbers of TNBC, HER2-positive, and ER-positive, different than their corresponding groups in breast cancer cell lines. This suggests that other cell phenotypes within tumors may modulate OR gene upregulation and that these phenotypes may be present in any tumor subtype.

Our results may be further analyzed to examine the ectopic expression of OR genes in breast tumors among cancer cells or other tumor resident or infiltrating cell types. Although breast cancer cell lines are not natural tumor cells, they provide models that mimic the tumor cell characteristics. The level of OR gene upregulation is generally lower in pure breast cancer cell lines compared to the OR upregulation levels in invasive breast carcinomas composed of multiple cell subtypes and derived from patient tumors. The elevated degree of upregulation level in breast tumors relative to cell lines potentially suggests a role for tumor microenvironmental characteristics as a supporting driver of OR dysregulation. Another hypothesis consistent with our observations is that non-cancer cells in the tumor, such as immune cells, may overexpress OR genes, perhaps under the dysregulated microenvironmental context created by the tumor. Each of these interesting hypotheses support further investigation.

## Conclusion

This study evaluates the transcript abundance of OR genes in human invasive breast carcinoma. OR2W3 is significantly upregulated among a large sub-population of these patients, in which gene signatures of invasion, cancer progression and basal-like subtype are abundant. Reduced probability of survival associated with OR2W3 upregulation among invasive breast carcinoma patients suggests that OR2W3 may be a potential breast cancer invasion marker. Further investigations are needed to characterize the mechanistic role(s) of OR genes in breast tumor progression.

## Materials and Methods

### Cases

The provisional breast invasive carcinoma study was selected from the TCGA repository including 1105 cases (March 2018). Among them, 960 cases with raw RNAseq, DNA amplification and survival probability data were selected and used in this study. RNAseq data (raw count files) of the 960 cases were downloaded from the Genomic Data Commons (https://gdc.nci.nih.gov/), the data portal for all the TCGA studies available through NCI. The survival and pathological information regarding each of the cases was obtained through the online tool cBioPortal^32,33^.

### Cancer cell line data

RNAseq level 3 data of all the cancer cell lines were retrieved from the CCLE database (http://www.broadinstitute.org/ccle)^34,35^. The format of the data files was processed RNAseq reads or fragments per kilobase of transcript per million of mapped reads (FPKM). The data corresponding only to the breast cancer cell lines were separated for further analysis.

### Cell culture

Breast cancer and normal mammary epithelial cell lines were obtained from American Type Culture Collection (ATCC, Manassas, VA). MDA-MB-231, MDA-MB-468, BT549, T47D and MFC7 human breast cancer cell lines were cultured in a growth medium consisting of high-glucose Dulbecco’s modified Eagle’s medium (DMEM), 10% FBS, 1% L-glutamine, and 1% penicillin/streptomycin (all materials from Fisher Scientific, Hampton, NH) at 37 °C and 5% CO_2_. MCF10A normal human mammary epithelial cells were cultured in mammary endothelial growth medium (MEGM) with 0.4% bovine pituitary extract (BPE), 0.1% hydrocortisone, 0.1% epidermal growth factor (hEGF), 0.1% insulin (Lonza, Walkersville, MD) with addition of 5% horse serum and 1% penicillin/streptomycin (Fisher Scientific) at 37 °C and 5% CO_2_. After two passages, all the cell lines were authenticated using short tandem repeat (STR) analysis (Genetica DNA Laboratories, Inc., Cincinnati, OH). Authentication was assessed by the percent match of each cell line to their corresponding ATCC database (Supplementary Data S1).

### mRNA isolation

Human breast cancer and normal mammary epithelial cells were cultured to 90% confluency and then collected by trypsinization. Total RNA was isolated from each sample using RNeasy tissue cell Mini-kit (Qiagen, Valencia, CA). To ensure high-purity RNA, any residual DNA in the samples was digested using 10 µL RNase-Free DNase set (Qiagen) per sample during the RNA purification procedure.

### RNAseq and data analysis

Libraries for RNAseq of cell lines were prepared from the total RNA isolates using PolyA tail selection (TruSeq mRNA library preparation kit, Illumina, San Diego, CA). The prepared libraries were sequenced using a HiSeq2500 (Illumina) as paired-end reads of 75 base-pair nucleotide lengths. The resulting RNAseq FASTQ files were obtained and the quality of the reads was assessed using FastQC v.0.11.0 high throughput sequence QC report tool by confirming that all base-pairs are in the high-quality (green) zone. The reads were aligned and mapped to the *Homo Sapiens* reference genome GRCh38.p10 using STAR read-aligner v.2.5.4^36^. SAMTools v.1.6 was used for sorting and indexing the BAM files^37,38^. Quantification of the raw counts in the mapped reads was performed using featureCounts (v1.5.3)^39^. The read counts for all entries was used to calculate FPKM. The data was presented as log_2_ transformed to normal distribution which were used for statistical analysis of variant abundance.

The RNAseq level 3 count files of TCGA patient data were obtained from paired-end reads of 76 base-pairs. The FPKM was calculated as above for all patients and subjected to hierarchical clustering for the genes of interest.

### Amplification data analysis

Chromosomal copy number alteration (CNA) of the invasive breast carcinoma patients were obtained from TCGA. The analyzed files of putative CNA by GISTIC 2.0 algorithm (Broad Institute) demonstrated allele amplification, heterozygosity or homozygosity^27^. The GISTIC algorithm assigns a putative CNA of 2 when the over-populated loci in genomes of the breast cancer patients are associated with high DNA amplification^40^. CNA values are also assigned as 1 for low-level gain, 0 for diploid, −1 for shallow loss of DNA (heterozygous deletion) and −2 for deep DNA deletion (homozygous deletion)^41^.

### Statistical analysis and calculations

To assess the potential significance of OR genes across all breast cancer patients, statistical calculations were performed on the FPKM data. Transcript abundance of the mRNAs was characterized as the log_2_ FPKM levels of their corresponding gene. The relative transcript abundance of the gene of interest in the breast tumor to its abundance in a normal mammary tissue (reference population, n = 4) was computed. The z-scores are computed as:

$${Z}_{ij}-score={X}_{ij}-{\bar{X}}_{i}/{\sigma }_{i}$$

where ${X}_{ij}$ is the abundance of gene i in patient j, $\overline{{X}_{i}}$ is the mean abundance of gene i in healthy tissues, and ${\sigma }_{i}$ is the standard deviation of gene i. Z-score indicates the level of gene i in any tumor away from the mean level in reference tissues, thus z-score >1 correlates with upregulation and z-score <−1 correlates with downregulation in that gene. In this study, OR genes with z-score above 2 were considered significantly upregulated whereas z-scores less than −2 were considered significantly downregulated. For each OR gene, the sum of upregulation is defined as:

$$Sum\,O{R}_{i}\,upregulation=\mathop{\sum }\limits_{j=1}^{m}\,{Z}_{ij}$$

where ${Z}_{ij}$ is the z-score of OR_i_ gene in patient j, and m is the total number of patients in the study with any OR gene upregulation. The number of cases with upregulated OR in which OR_i_ has a z-score >2 is defined by an indicator function as:

$${n}_{i}=\mathop{\sum }\limits_{j=1}^{m}\,[I({Z}_{ij})]\,:=\{\begin{array}{c}1\,if\,{Z}_{ij}\ge 2\\ 0\,if\,{Z}_{ij} < 2\end{array}$$

where [I(Z_ij_)] is the indicator function where it gives the value 1 if Z_ij_ is bigger than or equal to 2. In addition, the weighted OR upregulation is defined as:

$$Weighted\,O{R}_{i}\,upregulation={n}_{i}\times \mathop{\sum }\limits_{j=1}^{m}\,{Z}_{ij}$$

To identify the threshold of significance in the sum and weighted OR upregulation and number of cases with upregulated OR genes, we calculated the level of 95% confidence intervals where the upper-tail was used to indicate the higher levels than normal distribution. Standard deviation was used for variance, and p-value ≤ 0.05 represented the threshold of significance.

Supervised clustering was performed by Euclidean distance and Ward linkage method using the cluster package in R. For heatmap display, the supervised FPKM data of the OR genes were the input for the gplots package in R. In the heatmap, the y-data identifies the OR genes where each column represents an individual breast invasive carcinoma patient. Red color is indicative of relative high upregulation of that gene where blue indicates relative low upregulation. Statistical significance for gene abundance among sub-populations was calculated by one-way ANOVA. Similar significance measurement procedures were used for OR genes, Oncotype DX^®^ and PAM50 breast cancer-related genes. The overall survival data of the patients were obtained from TCGA, and the survival package in R was used for computing the survival probability. Significance in survival probability was determined by log-rank analysis (p-value ≤ 0.05). Preparation and statistical analysis for all the graphs were done using GraphPad Prism v7.

## Supplementary information

supplementary information

## References

[1] Noone, A. M. *et al*. (eds). SEER Cancer Statistics Review [Internet]. SEER Cancer Statistics Review, 1975–2015, National Cancer Institute. Bethesda, MD. Available from: https://seer.cancer.gov/csr/1975_2015/ (2017).

[2] Place, A. E., Jin, H. S. & Polyak, K. The microenvironment in breast cancer progression: Biology and implications for treatment. Vol. 13, Breast Cancer Research. (2011). 10.1186/bcr2912 PMC3326543 22078026

[3] Ma, X.-J. Dahiya, S. Richardson, E. Erlander, M. & Sgroi, D. C. Gene expression profiling of the tumor microenvironment during breast cancer progression. Breast Cancer Res [Internet]. 11(1):R7. Available from: http://www.ncbi.nlm.nih.gov/pubmed/19187537 (2009). 10.1186/bcr2222 PMC2687710 19187537

[4] Wang J, Rousseaux S, Khochbin S (2014). Sustaining cancer through addictive ectopic gene activation. Current Opinion in Oncology..

[5] Stemke-Hale K (2008). An integrative genomic and proteomic analysis of PIK3CA, PTEN, and AKT mutations in breast cancer. Cancer Res..

[6] Gray KA, Yates B, Seal RL, Wright MW, Bruford EA (2015). Genenames.org: The HGNC resources in 2015. Nucleic Acids Res..

[7] Buck L, Axel R (1991). A novel multigene family may encode odorant receptors: A molecular basis for odor recognition. Cell..

[8] Rouquier, S. *et al*. Distribution of olfactory receptor genes in the human genome. Nat Genet [Internet]. **18**(3):243–50. Available from: http://www.ncbi.nlm.nih.gov/pubmed/9500546 (1998). 10.1038/ng0398-243 9500546

[9] Buck LB (2000). The molecular architecture of odor and pheromone sensing in mammals. Cell..

[10] Spehr M (2003). Identification of a testicular odorant receptor mediating human sperm chemotaxis. Science (80-)..

[11] Aisenberg, W. H. *et al*. Defining an olfactory receptor function in airway smooth muscle cells. *Sci Rep*. 6 (2016). 10.1038/srep38231 PMC5131280 27905542

[12] Neuhaus EM (2009). Activation of an olfactory receptor inhibits proliferation of prostate cancer cells. J Biol Chem..

[13] Rodriguez M (2014). PSGR promotes prostatic intraepithelial neoplasia and prostate cancer xenograft growth through NF-kappaB. Oncogenesis..

[14] Morita R (2016). Olfactory receptor family 7 subfamily C member 1 is a novel marker of colon cancer-initiating cells and is a potent target of immunotherapy. Clin Cancer Res..

[15] Leja J (2009). Novel markers for enterochromaffin cells and gastrointestinal neuroendocrine carcinomas. Mod Pathol..

[16] Muranen Taru A, Greco Dario, Fagerholm Rainer, Kilpivaara Outi, Kampjarvi Kati, Aittomaki Kristiina, Blomqvist Carl, Heikkila Paivi, Borg Ake, Nevanlinna Heli (2011). Breast tumors from CHEK2 1100delC mutation carriers: genomic landscape and clinical implications. Breast Cancer Research.

[17] Choi, Y., Hur, C. G. & Park, T. Induction of Olfaction and Cancer-Related Genes in Mice Fed a High-Fat Diet as Assessed through the Mode-of-Action by Network Identification Analysis. *PLoS One*. **8**(3) (2013). 10.1371/journal.pone.0056610 PMC3608641 23555558

[18] Weber, L. *et al*. Olfactory Receptors as Biomarkers in Human Breast Carcinoma Tissues. Front Oncol [Internet]. 8. Available from: 10.3389/fonc.2018.00033/full (2018). 10.3389/fonc.2018.00033 PMC5818398 29497600

[19] Dowsett M (2010). Prediction of risk of distant recurrence using the 21-gene recurrence score in node-negative and node-positive postmenopausal patients with breast cancer treated with anastrozole or tamoxifen: A TransATAC study. J Clin Oncol..

[20] Tibshirani R, Hastie T, Narasimhan B, Chu G (2002). Diagnosis of multiple cancer types by shrunken centroids of gene expression. Proc Natl Acad Sci [Internet]..

[21] Parker JS (2009). Supervised risk predictor of breast cancer based on intrinsic subtypes. J Clin Oncol [Internet].

[22] Xu LL (2000). PSGR, a novel prostate-specific gene with homology to a G protein-coupled receptor, is overexpressed in prostate cancer. Cancer Res..

[23] Weng J (2006). PSGR2, a novel G-protein coupled receptor, is overexpressed in human prostate cancer. Int J Cancer..

[24] Sanz Guenhaël, Leray Isabelle, Dewaele Aurélie, Sobilo Julien, Lerondel Stéphanie, Bouet Stéphan, Grébert Denise, Monnerie Régine, Pajot-Augy Edith, Mir Lluis M. (2014). Promotion of Cancer Cell Invasiveness and Metastasis Emergence Caused by Olfactory Receptor Stimulation. PLoS ONE.

[25] Robinson, M. D. & Oshlack, A. A scaling normalization method for differential expression analysis of RNA-seq data. *Genome Biol*. **11**(3) (2010). 10.1186/gb-2010-11-3-r25 PMC2864565 20196867

[26] Maza E, Frasse P, Senin P, Bouzayen M, Zouine M (2013). Comparison of normalization methods for differential gene expression analysis in RNA-Seq experiments. Commun Integr Biol [Internet]..

[27] Mermel, C. H. *et al*. GISTIC2.0 facilitates sensitive and confident localization of the targets of focal somatic copy-number alteration in human cancers. *Genome Biol*. **12**(4) (2011). 10.1186/gb-2011-12-4-r41 PMC3218867 21527027

[28] Koboldt DC (2012). Comprehensive molecular portraits of human breast tumours. Nature..

[29] Shen Y (2015). LINC00472 expression is regulated by promoter methylation and associated with disease-free survival in patients with grade 2 breast cancer. Breast Cancer Res Treat..

[30] Stefansson OA (2015). A DNA methylation-based definition of biologically distinct breast cancer subtypes. Mol Oncol..

[31] Hao X (2017). DNA methylation markers for diagnosis and prognosis of common cancers. Proc Natl Acad Sci..

[32] Gao J., Aksoy B. A., Dogrusoz U., Dresdner G., Gross B., Sumer S. O., Sun Y., Jacobsen A., Sinha R., Larsson E., Cerami E., Sander C., Schultz N. (2013). Integrative Analysis of Complex Cancer Genomics and Clinical Profiles Using the cBioPortal. Science Signaling.

[33] Cerami E (2012). The cBio Cancer Genomics Portal: An open platform for exploring multidimensional cancer genomics data. Cancer Discov..

[34] Barretina J (2012). The Cancer Cell Line Encyclopedia enables predictive modelling of anticancer drug sensitivity. Nature..

[35] Cancer Cell Line Encyclopedia Consortium Genomics of Drug Sensitivity in Cancer Consortium. Pharmacogenomic agreement between two cancer cell line data sets. Nature [Internet]. 528(7580):84–7. Available from: http://www.nature.com/doifinder/10.1038/nature15736%5Cnhttp://www.ncbi.nlm.nih.gov/pubmed/26570998 (2015). 10.1038/nature15736 PMC6343827 26570998

[36] Dobin A (2013). STAR: Ultrafast universal RNA-seq aligner. Bioinformatics..

[37] Li H (2009). The Sequence Alignment/Map format and SAMtools. Bioinformatics..

[38] Li H (2011). A statistical framework for SNP calling, mutation discovery, association mapping and population genetical parameter estimation from sequencing data. Bioinformatics..

[39] Liao Y, Smyth GK, Shi W (2014). FeatureCounts: An efficient general purpose program for assigning sequence reads to genomic features. Bioinformatics..

[40] Beroukhim R (2007). Assessing the significance of chromosomal aberrations in cancer: Methodology and application to glioma. Proc Natl Acad Sci [Internet]..

[41] Ciriello G (2013). Emerging landscape of oncogenic signatures across human cancers. Nat Genet..

