# Supplementary material for: Olfactory receptor gene abundance in invasive breast carcinoma
Source: Sci Rep. 2019 Sep 24;9:13736. doi: 10.1038/s41598-019-50085-4 (PMC6760194; doi:10.1038/s41598-019-50085-4)
Supplement: Supplementary file 1 — supplementary information [file 41598_2019_50085_MOESM1_ESM.docx]

Olfactory receptor gene abundance in invasive breast carcinoma

Shirin Masjedi^1^, Laurence J Zwiebel^2^, Todd D Giorgio^1,3^

1 Department of Biomedical Engineering, Vanderbilt University

2 Department of Biological Sciences, Vanderbilt University

3 Department of Chemical and Biological Engineering, Vanderbilt University

Corresponding author: Todd Giorgio [todd.d.giorgio@vanderbilt.edu](mailto:todd.d.giorgio@vanderbilt.edu)

**Supplementary Information:**

**Table S1.** Cell line authentication with STR analysis on the breast cancer and mammary epithelial cell lines used in this study

| **Cell line** | **subtype** | **% match** | **Culture media** |
| --- | --- | --- | --- |
| MDA-MB-231 | Triple negative | 93.33 | high-glucose DMEM 10%FBS,1%Pen/Strep |
| MDA-MB-468 | Triple negative | 100 | high-glucose DMEM 10%FBS,1%Pen/Strep |
| MCF7 | luminal-A | 100 | high-glucose DMEM 10%FBS,1%Pen/Strep |
| BT549 | Triple negative | 91.67 | high-glucose DMEM 10%FBS,1%Pen/Strep |
| T47D | luminal-A | 100 | high-glucose DMEM 10%FBS,1%Pen/Strep |
| MCF10a | mammary epithelial | 100 | MEGM epithelial growth factors,1%Pen/Strep |


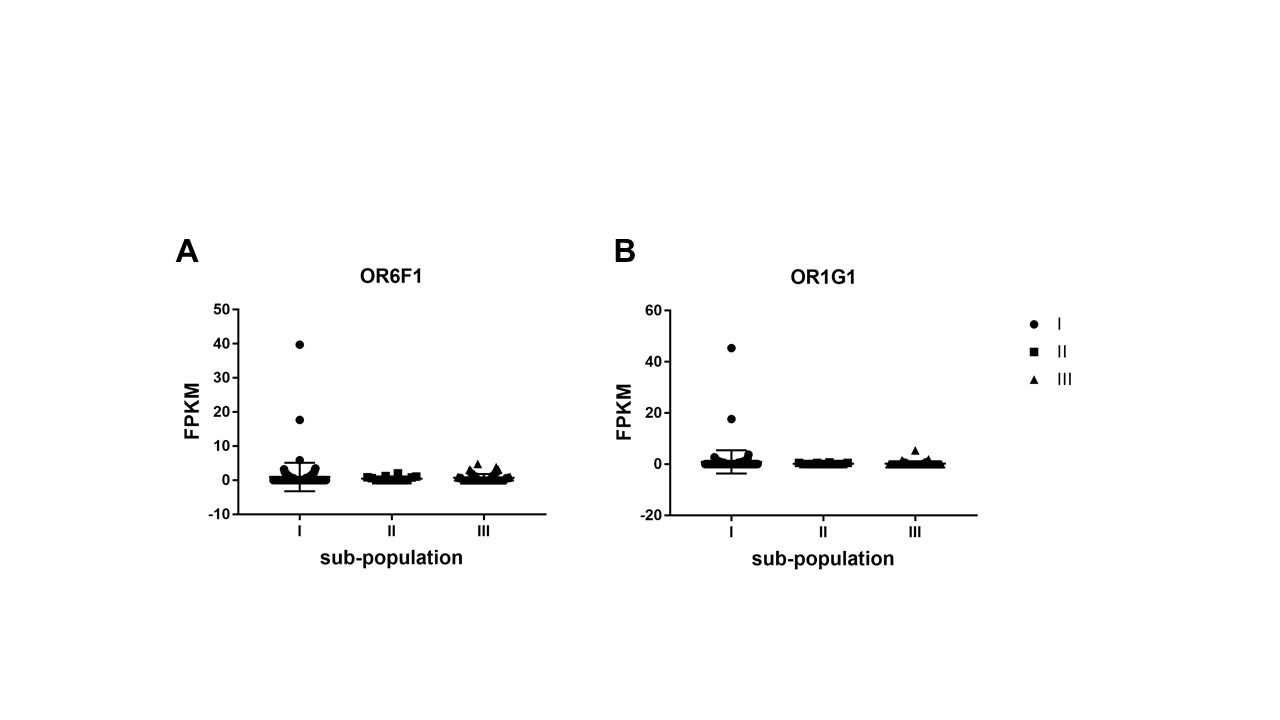


**Figure S2.** **A,** OR6F1 and **B,** OR1G1 are abundant in a few invasive breast carcinoma patients, but no correlation to a sub-population is observed.


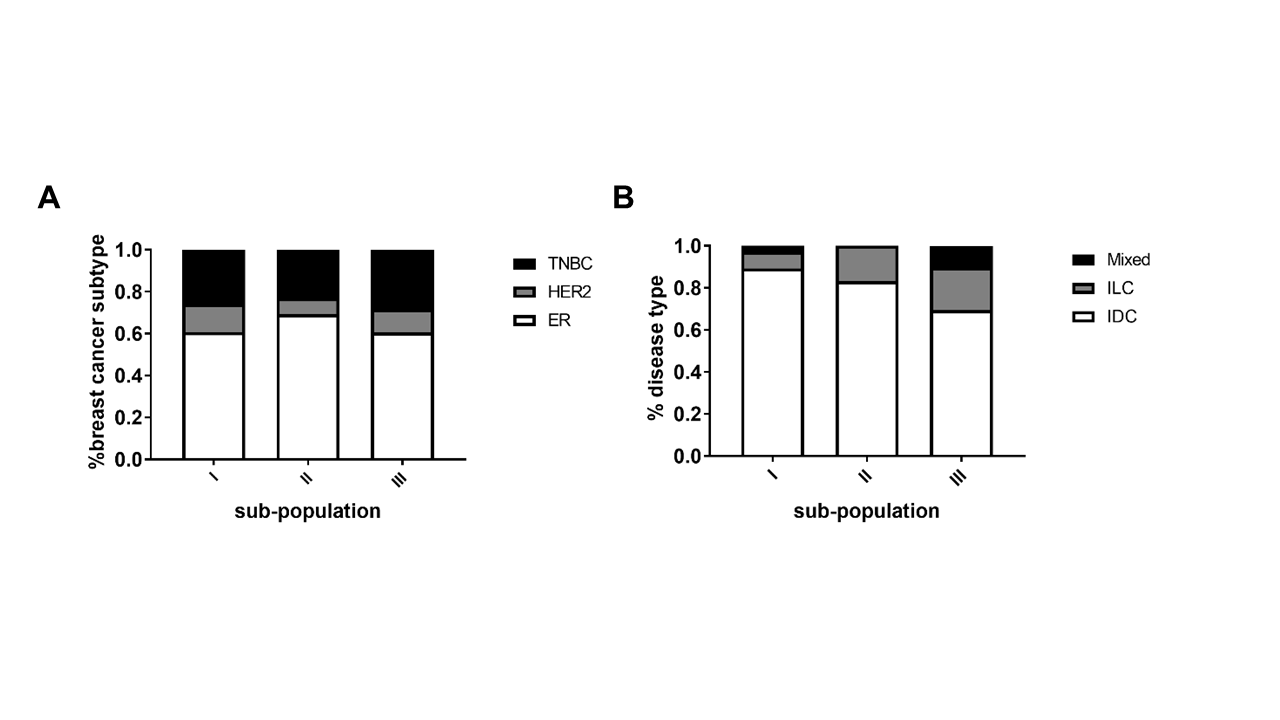


**Figure S3.** **A,** no significant difference in the distribution of ER-positive, HER-positive or TNBC, and **B,** of IDC, ILC or mixed morphological breast cancer types among the cases in sub-populations I, II and III with OR2B6, OR2T8 and OR2W3 upregulation respectively.


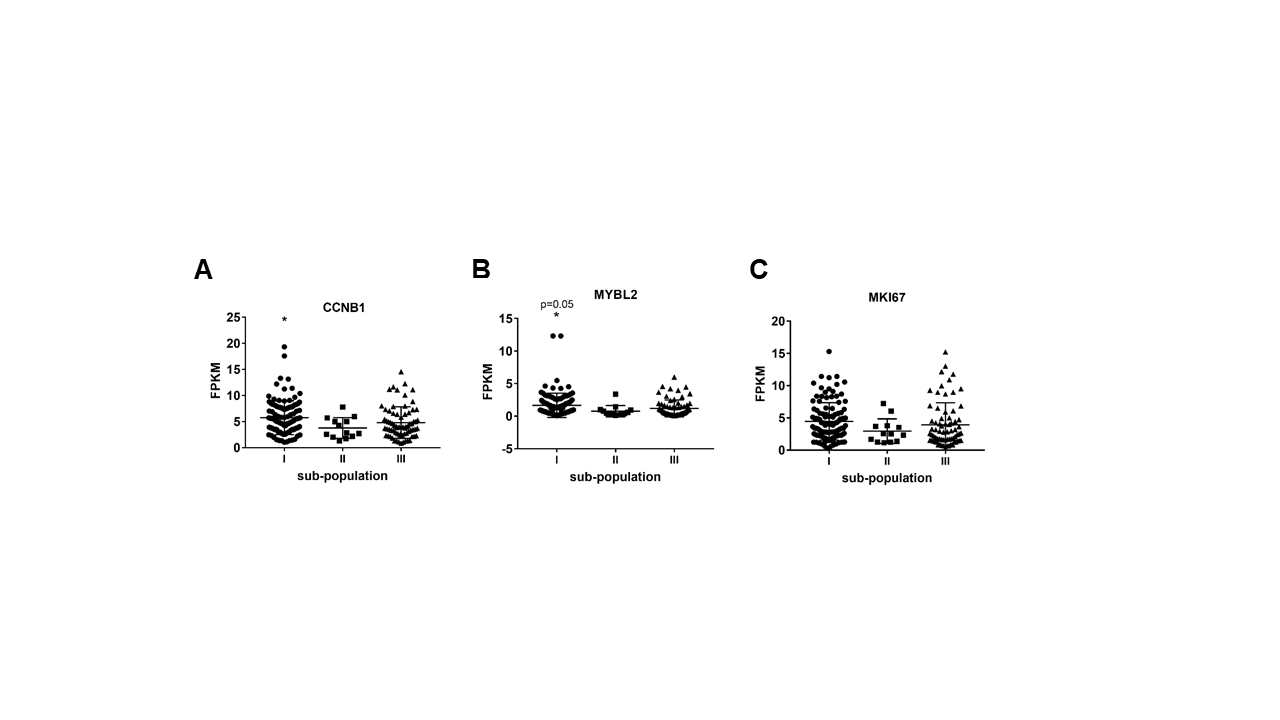


**Figure S4.** **A,** CCNB1, **B,** MYBL2 and **C,** MKI67 are significantly abundant among patients in sub-population I which are correlated with OR2B6 upregulation (p<0.05, one-way ANOVA).


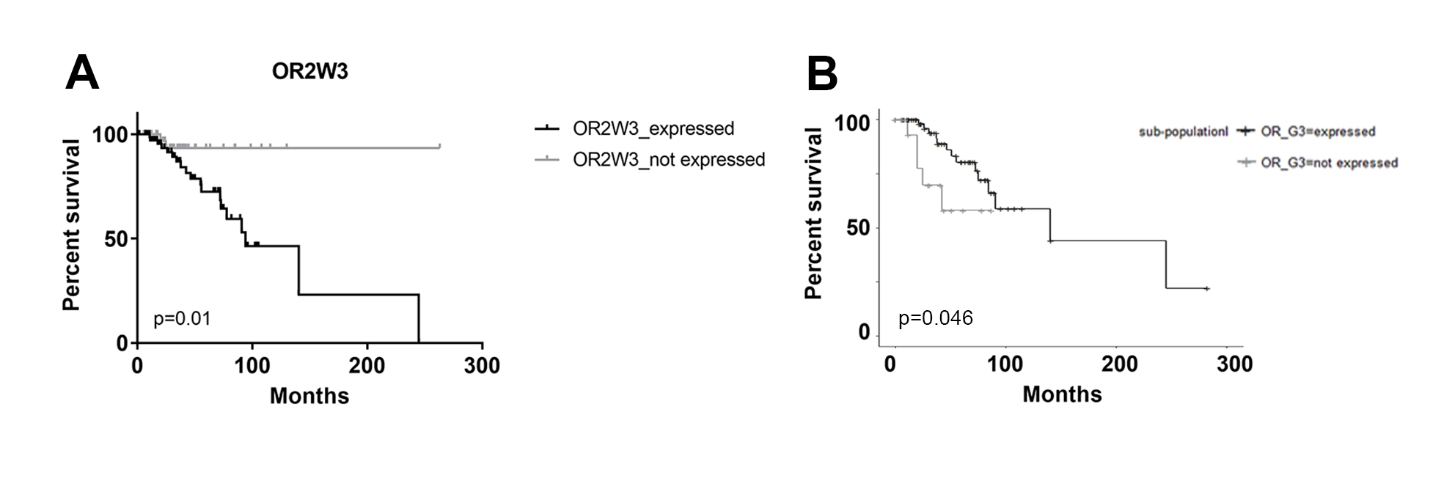


**Figure S5.** Kaplan-Meier plots of survival probability in breast invasive carcinoma patients. **A,** OR2W3 upregulation resulted in significantly lower survival probability after 250 months among breast invasive carcinoma patients (*p<0.05, log-rank test). **B,** Patients with OR group 3 gene expression have less survival probability versus patients not expressing OR group 3 after 250 months.


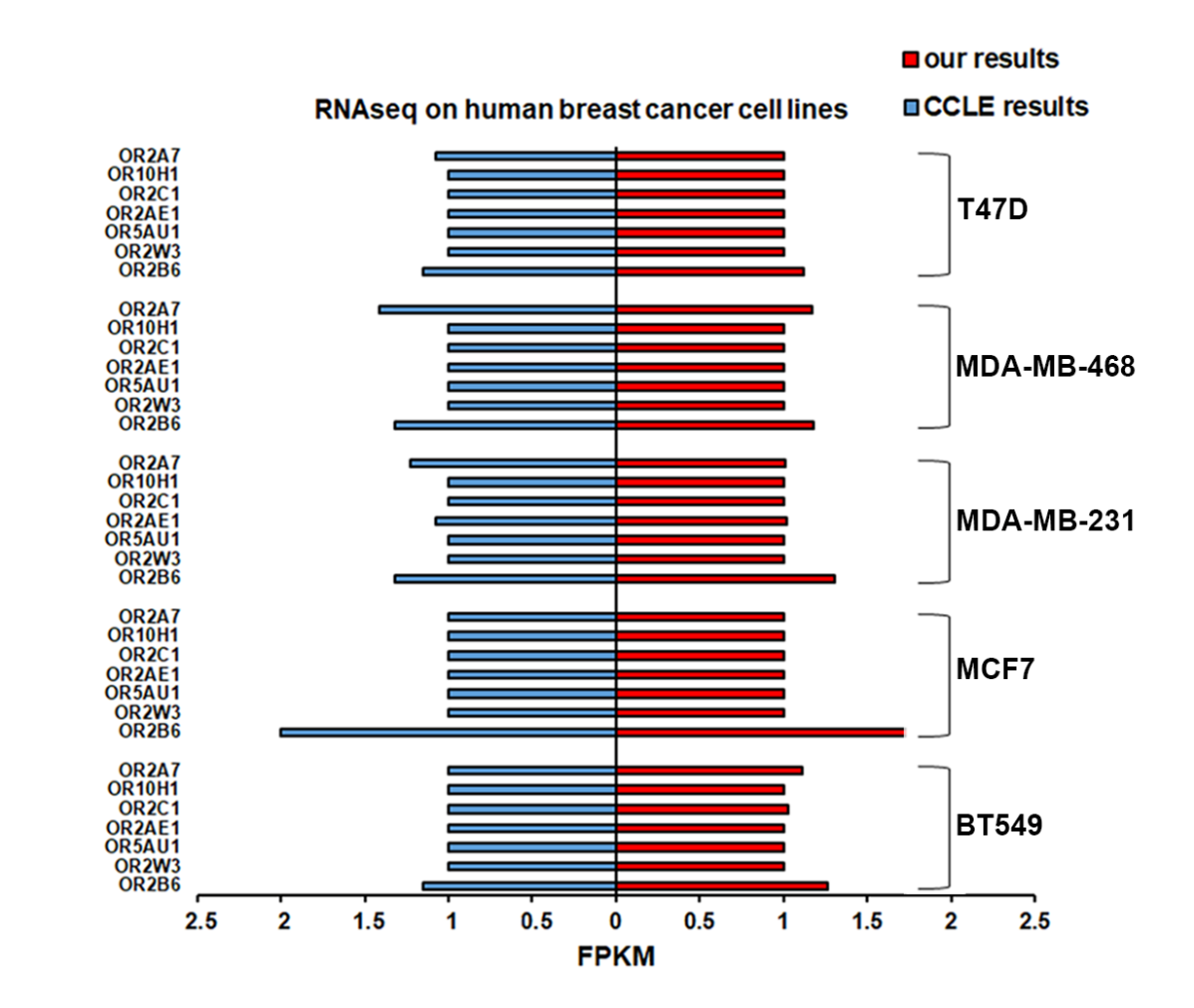


**Figure S6.** OR upregulation in human breast cancer cell lines based on RNAseq performed in our lab were comparable to the RNAseq results obtained from CCLE.
